# Structure of the ALS Mutation Target Annexin A11 Reveals a Stabilising N-Terminal Segment

**DOI:** 10.3390/biom10040660

**Published:** 2020-04-24

**Authors:** Peder A. G. Lillebostad, Arne Raasakka, Silje J. Hjellbrekke, Sudarshan Patil, Trude Røstbø, Hanne Hollås, Siri A. Sakya, Peter D. Szigetvari, Anni Vedeler, Petri Kursula

**Affiliations:** 1 Department of Biomedicine, University of Bergen, Jonas Lies vei 91, 5009 Bergen, Norway; plillebostad@gmail.com (P.A.G.L.); arne.raasakka@uib.no (A.R.); silje_johj@hotmail.com (S.J.H.); sudarshan.patil@uib.no (S.P.); trude.kr@gmail.com (T.R.); hanhol1404@gmail.com (H.H.); siriasakya@gmail.com (S.A.S.); peter.szigetvari@uib.no (P.D.S.); 2 Faculty of Biochemistry and Molecular Medicine & Biocenter Oulu, University of Oulu, Aapistie 7, 90220 Oulu, Finland

**Keywords:** annexin A11, crystal structure, solution structure, calcium binding, membrane binding, protein stability, folding, amyotrophic lateral sclerosis

## Abstract

The functions of the annexin family of proteins involve binding to Ca^2+^, lipid membranes, other proteins, and RNA, and the annexins share a common folded core structure at the C terminus. Annexin A11 (AnxA11) has a long N-terminal region, which is predicted to be disordered, binds RNA, and forms membraneless organelles involved in neuronal transport. Mutations in AnxA11 have been linked to amyotrophic lateral sclerosis (ALS). We studied the structure and stability of AnxA11 and identified a short stabilising segment in the N-terminal end of the folded core, which links domains I and IV. The crystal structure of the AnxA11 core highlights main-chain hydrogen bonding interactions formed through this bridging segment, which are likely conserved in most annexins. The structure was also used to study the currently known ALS mutations in AnxA11. Three of these mutations correspond to buried Arg residues highly conserved in the annexin family, indicating central roles in annexin folding. The structural data provide starting points for detailed structure–function studies of both full-length AnxA11 and the disease variants being identified in ALS.

## 1. Introduction

The annexin (Anx) family of Ca^2+^-binding proteins is widely distributed in eukaryotes, with twelve Anxs found in vertebrates [1,2,3]. All Anxs share multiple structural elements, most notably an evolutionarily conserved C-terminal Anx core. This core consists of four similar domains, each comprised of ~70 amino acids arranged into five α helices, termed A–E [1,4]. The only exception is AnxA6, which contains eight domains, most likely due to a fusion of duplicated *anx*A5 and *anx*A10 genes [5]. The five helices within each domain are connected via short loops, forming a right-handed superhelix. Helices A and B as well as helices D and E are oriented pairwise in an antiparallel fashion, while helix C is positioned as a bridge orthogonally to the others [4]. The loops connecting helix A to helix B and helix D to helix E contain the Ca^2+^ binding sites [1,6].

The Anxs are structurally distinct from the EF-hand and C2-domain Ca^2+^-binding proteins [2]. They reversibly bind negatively charged lipids in a Ca^2+^-dependent manner, which is a defining feature of this protein family [1]. The binding is mediated through the convex side of the core structure, coupled to a conformational change induced by Ca^2+^ binding [1,2]. Through lipid binding, Anxs can affect important proteolipid membrane properties, such as curvature and phase separation [7,8].

While the folded C-terminal core structure of the vertebrate Anxs is conserved, the structure of the N terminus varies greatly between different Anxs and determines their individual properties [1]. This region typically contains 10–30 residues, but AnxA7 and AnxA11 harbour unusually long N termini. AnxA11 has the longest N-terminal domain of all Anxs, with ~200 residues [9]. The N termini of AnxA7 and AnxA11 resemble each other, both being hydrophobic, enriched in Gly, Tyr, and Pro [10,11,12]. The Ca^2+^-dependent interaction of AnxA11 with phosphatidylserine changes the conformation of its N terminus [13]. Furthermore, the AnxA11 N terminus interacts with the apoptosis-linked gene-2 protein (ALG-2) [14] and S100A6 (calcyclin) [15].

Rabbit AnxA11 appears to exist as two isoforms; AnxA11A and AnxA11B [16]. Only the former binds S100A6 in the presence of Ca^2+^ via the region Gln49-Thr62. This region is absent in AnxA11B [16,17,18], suggesting isoform-specific functions. S100A6 binds to AnxA1 and AnxA2 in addition to AnxA11 [19,20], indicating functional overlap between these three proteins. According to the UniProt database [21], human AnxA11 is predicted to have two isoforms due to alternative splicing (UniProt ID P50995), leading to a truncation of 33 residues in the N terminus of the shorter form, while rat AnxA11 only exists as a single isoform corresponding to the longer human isoform (UniProt ID Q5XI77).

In certain cell types, AnxA11 is predominantly localised in the cytoplasm, while in others, it is mainly present in the nucleus [15,22]. It is likely that the A isoform is enriched in the nucleus, whereas the B isoform is specifically localised to the cytoplasm [16]. The N terminus was suggested to be responsible for the nuclear localisation [18,23]. In the nucleus, AnxA11 appears to be involved in events related to the cell cycle. It participates in cytokinesis, playing an essential role in the formation of the midbody. Cells lacking AnxA11 cannot complete cytokinesis and undergo apoptosis [24].

Expression levels of AnxA11 have been linked to various cancers [25,26,27,28,29,30]. Recent studies have revealed that mutations in AnxA11 play an important role in the development of the neurodegenerative disease amyotrophic lateral sclerosis (ALS) [8,31,32,33,34,35,36,37]. The molecular basis of these mutations is unclear, as an experimentally determined 3D structure of AnxA11 has been lacking.

Since the long N terminus of AnxA11 renders recombinant production of the protein challenging, N-terminally truncated recombinant forms were generated, and the crystal structure of one form, Δ188AnxA11, was determined. We show that a 4-residue sequence motif in the N terminus of the core structure, conserved in most Anxs, functions as a Velcro-like bridging segment and is important for the thermal stability of the Anx core structure. The currently known ALS mutations are analysed based on the crystal structure, providing a view into conserved features of the Anx core fold.

## 2. Materials and Methods

### 2.1. Cloning of Full-Length and N-Terminally Truncated Forms of AnxA11

In order to obtain full-length rat AnxA11, reverse transcriptase-polymerase chain reaction (RT-PCR) was performed using total RNA isolated from PC12 cells by the Trizol method [38]. The cDNA of AnxA11 was obtained using the iScript kit (Bio-Rad, Hercules, CA, USA) according to the manufacturer’s instructions and the forward primer 5′-ATCCGGCCATGGGTATGAGCTATCCAGGCTATCCAC-3′ (*Nco*I restriction site is underlined) and the reverse primer 5′-ATCCGGGGTACCTCAGTCGTTGCCACCACAGATC-3′ (*Acc*65I restriction site is underlined). The *anx*A11 cDNA was digested with *Nco*I and *Acc*65I, purified after separation in a 1% agarose gel, and ligated into the same restriction sites of the pETM10 vector (a kind gift from Gunter Stier, Heidelberg). Two truncated versions of AnxA11 were generated by PCR using the pETM10 plasmid harbouring rat full-length *anx*A11 cDNA as a template and using the same reverse primer as for full-length AnxA11. The forward primers were 5′-ATCCGGCCATGGGTAGAGGCACCATCA-3′ and 5′-ATCCGGCCATGGGTACCGATGCTTCTG-3′ (the *Nco*I restriction site is underlined), resulting in Δ188AnxA11 (starting with 189-RGTI) and Δ192AnxA11 (starting with 193-TDAS), respectively. Both constructs code for the tag sequence MLHHHHHHPMG at the N terminus. The cDNAs were ligated into the pETM10 vector after digestion of PCR fragments with *Acc*65I and *Nco*I. Constructs were verified by DNA sequencing.

### 2.2. Expression and Purification of Full-Length and N-Terminally Truncated Forms of AnxA11

*E. coli* BL21(DE3) cells were transformed with AnxA11 expression plasmids. The bacteria were grown in 5 mL lysogeny broth (LB) medium with 50 µg/mL kanamycin until an OD_600_ of 0.6, after which 1 mM isopropyl β-D-1-thiogalactopyranoside (IPTG) was added to induce protein expression at +25 °C for 4 h or at +15 °C overnight (ON), to determine optimal expression conditions. At the end of protein expression, 1 mL of the culture was withdrawn and centrifuged at 2000× *g* for 10 min at +4 °C. The supernatant was discarded, and the pellet was resuspended in 500 µL lysis buffer (50 mM Na_2_HPO_4_, 500 mM NaCl, 5% (*v*/*v*) glycerol, 10 mM imidazole) containing 0.5 µg/mL DNase I (Sigma, St. Louis, MO, USA), 0.25 µg/mL RNase A (Sigma, St. Louis, MO, USA), and cOmplete EDTA-free protease inhibitor cocktail (Roche, Basel, Switzerland). The samples were sonicated on ice, whereafter 100-µL aliquots (total lysate) were withdrawn. Additional 100-µL aliquots of the lysates were transferred into new tubes and centrifuged at 21,000× *g* for 15 min at +4 °C. The supernatant was withdrawn. The pellet was resuspended in 100 µL lysis buffer. After addition of denaturation buffer, 20 µL of the protein samples were separated on 4–20% SDS-PAGE (Mini-PROTEAN TGX Precast Protein Gels, Bio-Rad, Hercules, CA, USA) at 20 mA and 250 V as the limiting voltage.

For large-scale purification, the above procedure was repeated using 300 mL LB medium. The full-length (FL) and N-terminally truncated AnxA11 were induced at +25 °C for 4 h, while FL-AnxA11 was also expressed ON at +15 °C. The cells were collected by centrifugation at 4000× *g* for 15 min. The pellets were frozen at −80 °C in lysis buffer before the addition of 0.5 µg/mL DNase I, 0.25 µg/mL RNase A, and cOmplete EDTA-free protease inhibitor cocktail (Roche, Basel, Switzerland). Subsequently, cells were broken by sonication, and the lysates were centrifuged at 16,000× *g* for 30 min at +4 °C. All purification steps were performed at +4 °C.

We took advantage of an N-terminal His-tag to purify AnxA11, which reduces degradation at the N terminus [13]. Purification using Co^2+^-NTA gave higher yields than Ni^2+^-NTA resin. The lysate supernatant was loaded onto a Co^2+^-NTA agarose column and incubated for 30 min with gentle rotation. Subsequently, the proteins on the Co^2+^ resin were washed with equilibration buffer (50 mM Na_2_HPO_4_, 0.3 M NaCl, 10 mM imidazole; pH 8) and high-salt buffer (50 mM Na_2_HPO_4_, 1 M NaCl, 10 mM imidazole; pH 8), both buffers containing EDTA-free protease inhibitors. Elution buffer (50 mM Na_2_HPO_4_, 0.3 M NaCl, 250 mM imidazole; pH 8) containing EDTA-free protease inhibitors was used to elute the proteins. After adding EGTA to a final concentration of 2 mM, the eluates were quickly transferred onto PD-10 columns for a buffer exchange to 20 mM Tris (pH 8). All recombinant forms of AnxA11 were subjected to size exclusion chromatography using a Superdex 75 or 200 Increase 10/300 GL column (GE Healthcare, Chicago, IL, USA). Protein concentration was determined by absorbance at 280 nm (using molecular masses of 55513, 36828, and 36401 Da and sequence-based extinction coefficients of 42750, 13410, and 13410 M^−1^ cm^−1^ for full-length AnxA11, Δ188AnxA11, and Δ192AnxA11, respectively). The protein size and purity were confirmed by SDS-PAGE and Coomassie Brilliant Blue staining.

### 2.3. Circular Dichroism Spectroscopy

A Jasco J-810 spectropolarimeter (Jasco, Hampshire, UK) with a Peltier temperature control unit was used for far-UV circular dichroism (CD) spectroscopy. Melting curves were recorded from +25 to +75 °C at 222 nm, at a heating rate of 40 °C/h, to determine the thermal transition temperature (T_m_). T_m_ was estimated in GraphPad Prism 7 (GraphPad, San Diego, CA, USA) using four-parameter logistic regression.

Synchrotron radiation CD (SRCD) data were acquired from 0.5 mg/mL Δ188AnxA11 and Δ192AnxA11 in 20 mM Tris-HCl, pH 8.0, on the AU-CD synchrotron beamline at ASTRID2 (ISA, Aarhus, Denmark). Samples containing 1 mM CaCl_2_ were included in addition to CaCl_2_-free samples to detect Ca^2+^-induced changes in the secondary structure content. Closed 100-µM circular cells were used (Suprasil, Hellma Analytics, Müllheim, Germany). Spectra were recorded from 170 to 280 nm at +10 °C. Baselines were subtracted, and CD units were converted to Δε (M^−1^ cm^−1^) in CDtoolX 1.06 [39]. The spectra were truncated based on the HT voltage and the CD spectrum noise levels.

### 2.4. Small-Angle X-Ray Scattering

SAXS data were collected from 0.41–2.96 mg/mL Δ188AnxA11 and Δ192AnxA11 in 20 mM Tris-HCl on the EMBL/DESY P12 beamline, PETRA III (Hamburg, Germany) [40] (Figure S1). Protein samples at 0.7–1.0 mg/mL were then studied with 0–1 mM CaCl_2_ to follow potential oligomerisation. Monomeric bovine serum albumin (66.5 kDa) was used as a molecular weight standard. Data were processed and analysed using the ATSAS package 2.8.4 [41], and GNOM 5.0 was used to calculate distance distribution functions [42]. Theoretical scattering of the crystal structure was calculated and fitted against the SAXS data using CRYSOL 2.8.3 [43]. Ab initio modelling was performed using GASBOR 2.3i [44]. SUPCOMB 2.3 [45] was used for crystal structure and ab initio model superposition. SAXS data collection, processing, and fitting parameters are presented in Table 1.

### 2.5. Protein Crystallisation, Data Collection, and Structure Determination

Sitting-drop vapour diffusion crystallisation experiments were set up in 96-well format. X-ray diffraction data were collected on the EMBL P13 beamline, PETRA III (Hamburg, Germany) [46] from a crystal grown in 100 mM Bis-tris (pH 5.5), 3 M NaCl at +20 °C; the drop contained 150 nL of 12.7 mg/mL Δ188AnxA11 and 150 nL of reservoir solution. The crystal was cryoprotected by adding a solution containing 25% glycerol and 75% reservoir solution directly onto the crystallisation drop. Upon mounting, the crystal was flash-frozen in liquid N_2_, and it was kept at 100 K during X-ray diffraction data collection.

Diffraction data were processed with XDS [47], and the structure was solved by molecular replacement in Phaser 2.8.3 [48] with PDB entry 1ANN (bovine AnxA4) as a search model [49]. Refinement was carried out using phenix.refine 1.18 [50], and model building with coot 0.8.9.1 [51]. The structure quality was assessed with MolProbity [52]. The data processing and refinement statistics are presented in Table 2.

For structure analysis, the programs PyMOL 2.3.2 [53] and its APBS [54] plugin were used. The refined AnxA11 crystal structure, along with the experimental structure factors, was deposited at the PDB with the entry code 6TU2.

## 3. Results and Discussion

### 3.1. Solubility Assays, Large-Scale Expression, and Purification of the N-Terminally Truncated Forms of AnxA11

**Figure 1:**
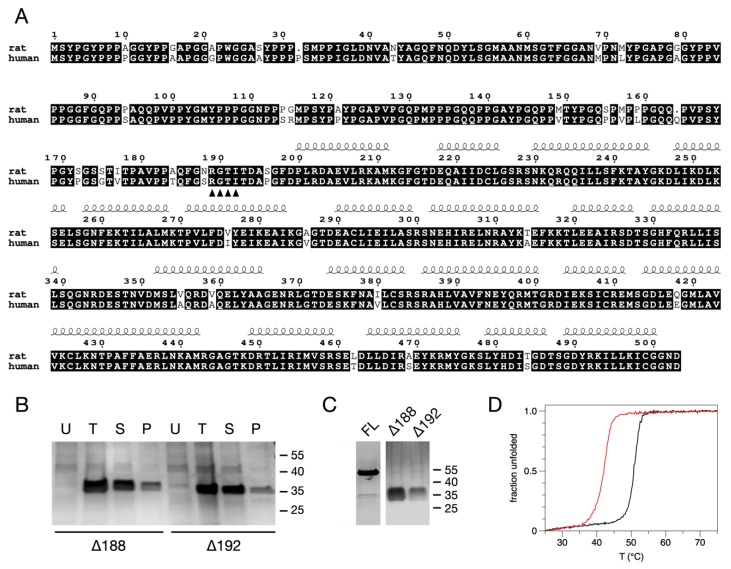
The solubility, purification, and stability of N-terminally truncated forms of rat AnxA11. (**A**) Sequence alignment between rat and human AnxA11. (**B**) Uninduced (U), total (T), supernatant (S), and pellet (P) fractions of bacterial lysates containing Δ188AnxA11 or Δ192AnxA11 as indicated. (**C**) 10 µL of FL-AnxA11 and the N-terminally truncated forms Δ188AnxA11 and Δ192AnxA11, purified using Co^2+^-resin. Molecular mass standards are indicated to the right. Proteins were separated in 10% SDS-PAGE gels and Coomassie-stained. (**D**) CD-monitored thermal disruption of α-helicity of 20 μM Δ188AnxA11 (black) and Δ192AnxA11 (red).

We studied recombinant rat AnxA11, which is nearly identical to the human AnxA11 (Figure 1A). Induction was performed at +25 °C for 4 h and at +15 °C ON to determine optimal expression conditions. There was little or no difference in yield between the two modes of expression. FL-AnxA11 was consistently more challenging to express than its truncated forms, yielding remarkably low concentrations, and it was notoriously prone to aggregation, as reported previously [13]. The solubility of the truncated forms of AnxA11 in bacterial lysates was assessed after centrifugation at 21,000× *g* by comparing samples from the total bacterial lysate with the corresponding supernatant and pellet by SDS-PAGE (Figure 1B). Two N-terminally truncated versions of AnxA11 were designed, cloned, expressed, and purified, namely, Δ188AnxA11 and Δ192AnxA11. Previously, we noticed by sequence alignment that a segment of ~10 residues in the N terminus preceding the first α helix of domain I is highly conserved in Anxs [55]. In addition, the N-terminal residues that immediately precede the core domain have been suggested to stabilise the overall structure of AnxA2 [55,56].

In contrast to FL-AnxA11, both Δ188AnxA11 and Δ192AnxA11 were mainly soluble (Figure 1B). FL-AnxA11 as well as the N-terminally truncated Δ188AnxA11 and Δ192AnxA11 were purified by affinity chromatography on Co^2+^-resin, which greatly improved the purity of the recombinant proteins compared to Ni^2+^-based chromatography. The N-terminal truncations resulted in a marked enhancement in expression, giving a five-to-tenfold increase in yield compared to FL-AnxA11 (Figure 1C). This suggests that the N terminus is responsible for the aggregation of the full-length protein. This may be related to the observed phase separation phenomena linked to the N-terminal segment [8] or misfolding of the protein in bacterial cells.

### 3.2. Thermal Stability of the N-Terminally Truncated Δ188AnxA11 and Δ192AnxA11

To address thermal stability, ellipticity at 222 nm was monitored as a function of temperature for Δ188AnxA11 and Δ192AnxA11. The thermal transition temperatures were estimated from the inflection points of the melting curves (Figure 1D). The T_m_ of FL-AnxA11 was earlier reported to be +45 °C [13]. The T_m_ of Δ188AnxA11 showed a T_m_ of +50.76 ± 0.02 °C, while that of Δ192AnxA11 was substantially lower (+41.87 ± 0.02 °C). This difference highlights the importance of the four amino acid residues 189-RGTI-192 in thermally stabilising the core structure of AnxA11. Cooperative unfolding of the two truncated forms of AnxA11 remained largely unchanged but decreased slightly in Δ192AnxA11. This increased thermal stability of Δ188AnxA11 compared to Δ192AnxA11 may explain why we obtained crystals of Δ188AnxA11, but not of the other N-terminally truncated form. The dramatic difference in T_m_ is necessarily accounted for by at least one of the four additional residues present in Δ188AnxA11 being involved in the stabilising of the core structure.

### 3.3. The Crystal Structure of AnxA11

**Figure 2:**
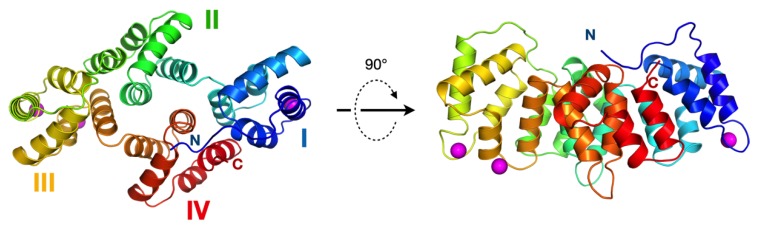
Crystal structure of the AnxA11 core domain. The four subdomains are numbered and coloured from blue to red. The N and C termini are indicated, and bound Ca^2+^ ions are shown in magenta. Shown is one monomer of the three in the asymmetric unit.

Using Δ188AnxA11, we solved the crystal structure of the rat AnxA11 core domain. The asymmetric unit contains three molecules of AnxA11, and the structure presents the canonical Anx core fold. The Ca^2+^ binding sites are partially occupied, whereby two monomers have three Ca^2+^ ions bound, and one monomer has four (Figure 2). As is typical for the Anxs, all Ca^2+^ binding sites are on one face of the molecule, promoting calcium-dependent lipid membrane binding (Figure 2).

**Figure 3:**
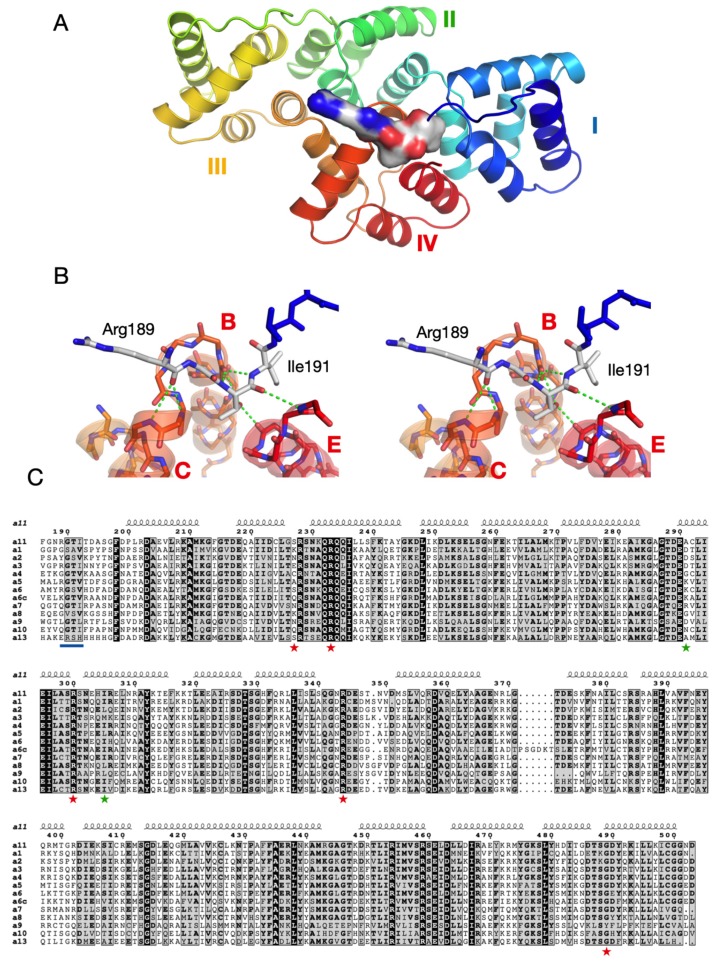
Rat annexins and conservation of the RGTI motif. (**A**) The N-terminal Velcro-like bridging segment is shown in surface mode, bound to domain IV. (**B**) Stereo view of the hydrogen bonding interactions of the motif with three helices in domain IV. (**C**) Sequence alignment of all rat Anxs. The N-terminal motif is underlined in blue, and the mutations (red) and variants (green) linked to ALS are shown with asterisks. A6 and A6c correspond to the N- and C-terminal halves of AnxA6.

The substantial difference in thermal stability induced by the four N-terminal residues of the Δ188AnxA11 construct implies an important structural element at this position. This segment, ~10 residues before the AnxA11 domain I helix A, is central in the folded structure, making interactions with the termini of several helices of the Anx fold, and effectively enabling a tight contact between the N terminus of domain I and the C terminus of domain IV (Figure 3A,B).

Comparisons with other Anxs show that the segment 189–192 is a conserved motif in the Anx family. The only Anx that does not contain the motif at the sequence level is AnxA13; conservation is weak for AnxA1 and AnxA8. The 4-residue segment forms conserved interactions with the ends of helices in domain IV (Figure 3B). These interactions are mainly comprised of main-chain hydrogen bonds, which explains the mode of conservation and the importance of the Gly residue in the motif. Interestingly, it has been proposed that AnxA11 is the common ancestor of all mammalian Anxs [57], except for AnxA7 and AnxA13—the two phylogenetically oldest Anxs [58]. Thus, the AnxA11 core domain has the highest similarity to the other mammalian Anxs, acting as a sort of prototype [59].

**Figure 4:**
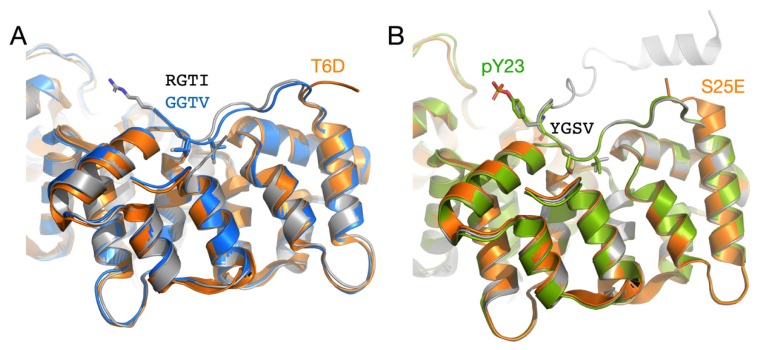
Regulation of the RGTI motif via phosphorylation in other annexins. (**A**) Comparison of AnxA11 (grey) with AnxA4 (blue) [49]. The structure of the phosphomimicking AnxA4 T6D mutant [61] is shown in orange and has an open conformation. (**B**) Three states of AnxA2 [56]. The nonphosphorylated protein is in grey, AnxA2 phosphorylated on Tyr23 in green, and the open conformation of S25E in orange.

The PhosphoSite database lists a number of phosphorylation sites for Anxs, most of which come from high-throughput assays [60]. AnxA11 is phosphorylated, but the exact sites remain unknown [15]. Interestingly, the well-characterised Tyr23 and Ser25 phosphorylation sites of AnxA2 lie within the 4-residue bridging region (Figure 3C). Phosphorylation at Tyr23 and Ser25 results in a closed or open conformation, respectively, of AnxA2 [56]. Similarly, a phosphomimicking mutation in Thr6 of AnxA4 results in an opening of the structure [61], hinting at a general mechanism of Anx conformation regulation through phosphorylation of the RGTI motif. A comparison of the structures of AnxA11, AnxA2, and AnxA4 is presented in Figure 4. Of other Anxs, AnxA6 has a phosphorylation site in the bridging segment [62]. These findings can be understood based on the conserved structure of the observed bridging segment. The regulation of this segment through post-translational modification may be a general means to regulate overall conformation and dynamics of Anxs, possibly resulting in altered binding to functional ligands, such as RNA or membranes.

**Figure 5:**
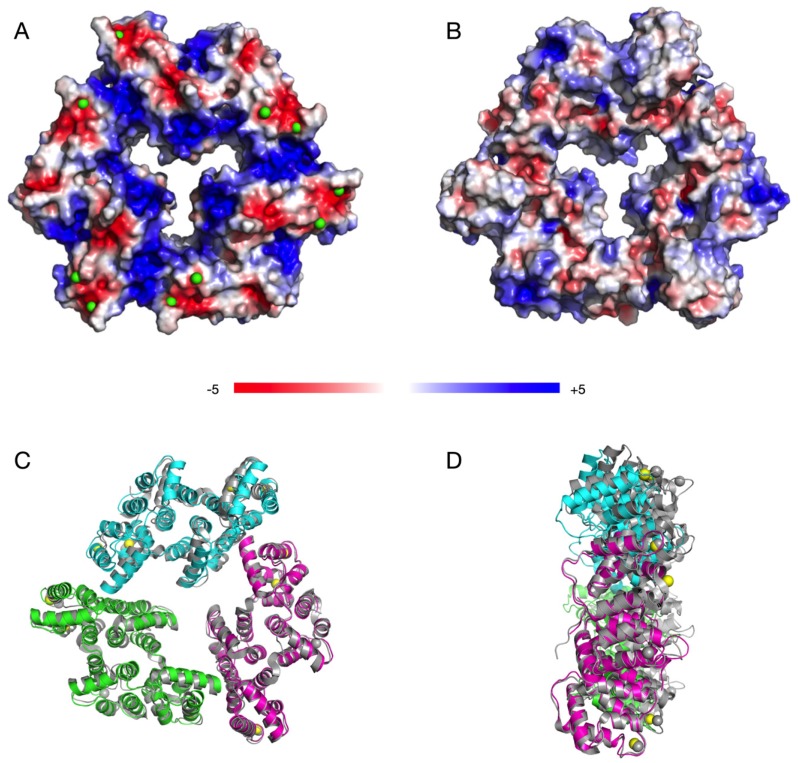
Trimeric assembly of AnxA11 in the crystal. (**A**) AnxA11 trimer surface coloured with electrostatic potential. The view from the Ca^2+^-binding face. Ca^2+^ ions are shown as green spheres. The negative sites (red) are the locations for Ca^2+^ binding, and their neutralization will make the surface very positive, enabling lipid membrane binding. (**B**) The opposite face of AnxA11 is relatively featureless. (**C**) Superposition of AnxA11 (coloured) with the AnxA5 trimer (gray) [64]. Superposition was done based on one monomer (bottom right) to better highlight possible differences in assembly. The view is the same as in (**B**). (**D**) Side view of the superimposed AnxA11 and AnxA5 trimers.

The crystal structure of AnxA11 presents trimers very similar to those formed by AnxA5 (Figure 5), and hence, could be expected to interact with membranes in a similar manner. In the crystal, the trimers are arranged in layers, with similar contacts as observed for AnxA4 and AnxA5 between the trimers. Similarly to other Anx assemblies, the Ca^2+^ binding sites of the AnxA11 trimer are all on the same face, which would be in direct contact with the membrane (Figure 5A). The membrane-facing surface in the trimer gains a positive charge potential upon Ca^2+^ binding, while the opposite side is relatively featureless (Figure 5B). This observation further highlights the common function of the Anx core structure in binding membrane surfaces, while the N-terminal domain is responsible for most functional differences between the Anxs [1]. AnxA5 has been the most studied Anx on membrane surfaces, on which it forms a crystal-like lattice of trimers [63,64,65,66]. The trimer arrangement of AnxA11 in the crystal closely resembles that of AnxA5 in crystals and on membranes (Figure 5C,D) [63,64,65,66]. Notably, similarly to AnxA11, AnxA4 and AnxA5 are monomeric in solution [67,68,69].

One might assume that AnxA11 would arrange on a membrane much like it does in the crystal; the N-terminal segments would then point away from the membrane. This is in line with the observations that AnxA11 induces membrane curvature [7], as well as the suggestion that the N terminus of AnxA11 binds to RNA, while the core structure binds to lipids in lysosomes for long-distance co-transport in neurons [8]. The RNA binding site in AnxA2 has been shown to reside within domain IV [70]. The binding dynamics were recently investigated; AnxA2 binds strongly to an RNA 8-mer (5′-GGGGAUUG-3′) with a K_d_ of 30 nM [71]. The RNA binding sites in AnxA11 have not been determined.

### 3.4. Structure-Based Analysis of ALS Disease Mutations

The crystal structure of rat AnxA11 allows one to analyse the structural basis of AnxA11 mutations linked to amyotrophic lateral sclerosis (ALS) [35] in humans. The sequence identity between full-length rat and human AnxA11 is 93.3%, being 96.5% for the core structure. The identification of AnxA11 as an ALS gene is rather recent, and mutations are being continuously identified. Thus, it is safe to assume that more ALS mutations will be identified in the future. We studied the mutations thus far linked to ALS with respect to potential structural implications. Many of the mutations, including the best-characterised D40G [35], lie in the N-terminal extension, which is not present in the crystallised construct. The mutations in this region could affect protein–protein interactions, protein localisation, phase separation, or binding to mRNA [8]. More interesting for the current structural study, however, are the mutations identified within the Anx core domain.

**Figure 6:**
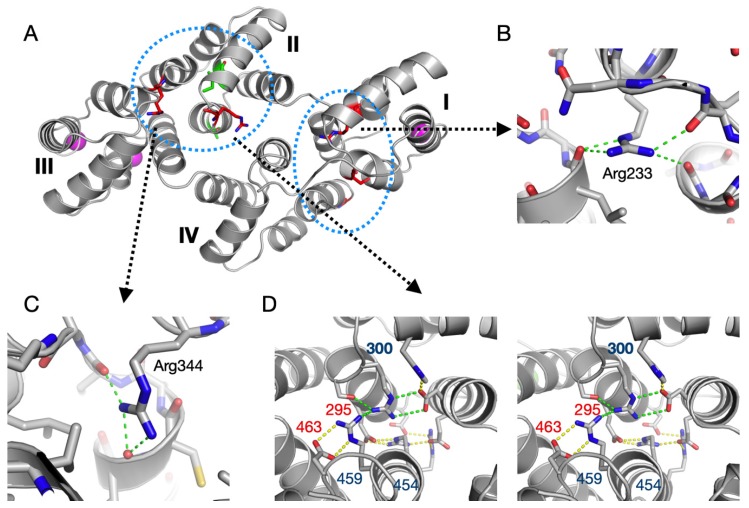
Location of ALS mutations and variants in the AnxA11 structure. (**A**) The five ALS mutations (see text) are highlighted in red and the two rare variants in green. The two 3D clusters of mutations/variants are shown with blue dashed circles. One cluster lies in domain II, possibly affecting folding and/or interactions with domain III. The other cluster is at the interface between domains I and IV, i.e., the N and C terminus of the AnxA11 core structure. (**B**) Arg233 (corresponding to the human R235Q mutation) makes four contacts to carbonyl groups at the C-terminal end of domain I helices B and E. With its buried positive charge, Arg233 interacts with the negative helix dipole from both helices. (**C**) Arg344 (corresponding to R346C) similarly caps helix B of domain III. (**D**) An extended salt bridge network exists between domains II (top) and IV (bottom) (stereo view). Arg300 (corresponds to R302C) is central in this network. The charged residues with numbers indicated are fully conserved in all rat Anxs (Arg, blue; acidic, red).

Five mutations have thus far been described in ALS patients within the AnxA11 core: S229R, R235Q, R302C, R346C, and G491R; in addition, G189E is located two residues before the start of the crystallised rat AnxA11 construct [35,37]. In the 3D structure, the AnxA11 mutations can be observed clustered at two locations (Figure 6A). Interestingly, three of these mutations involve a buried Arg residue, and a close look at the alignment reveals that these Arg residues are fully conserved in all rat Anxs. The remaining two mutations replace an exposed, non-conserved, small residue with an Arg. Details of the environment of each of the three conserved Arg residues are shown in Figure 6B–D. In brief, two of them coordinate carbonyl groups at C termini of helices, and one is involved in a conserved extended buried network of salt bridges between domains II and IV. These locations suggest that the ALS mutations involving these conserved Arg residues will have effects on AnxA11 folding and/or stability.

∆∆G predictions for the five mutations on the SDM server [72] indicate decreased stability for all mutations except S229R. Hence, the affected residues may be important for correct folding and/or stability of AnxA11. Recent functional characterisation of the AnxA11 ALS mutation R235Q shows that the mutant protein is prone to aggregation and sequesters FL-AnxA11. This in turn abolishes the binding to calcyclin [35]. In addition, two rare missense variants, A293V and I307M, were recently discovered in the Chinese Han population [34]. These residues are located in domain II (Figure 6).

The N-terminal tail of AnxA11 has a unique amino acid composition, reminiscent of disordered proteins able to undergo liquid–liquid phase separation (LLPS) [73]. It has the hallmarks of an extended segment poised for π-π interactions, which are central in LLPS [74]. Indeed, the AnxA11 N-terminal region was recently shown to promote LLPS; its N terminus binds to RNA granules, which do not contain membranes, while the core structure binds to lipids in membranes [8]. The effects of this phase separation on the stability of the AnxA11 core domain are currently unknown. Furthermore, ALS-associated mutations in both the N terminus (D40G) and C terminus (R346C) of AnxA11 were shown to promote phase transition from liquid–liquid droplets to more stable gel-like states within AnxA11 droplets and in addition impair the reversal of the states. As a consequence, these mutations disrupt the function of AnxA11 to tether RNA granules with lysosomes [8].

### 3.5. The Solution Behaviour of the AnxA11 Core

**Figure 7:**
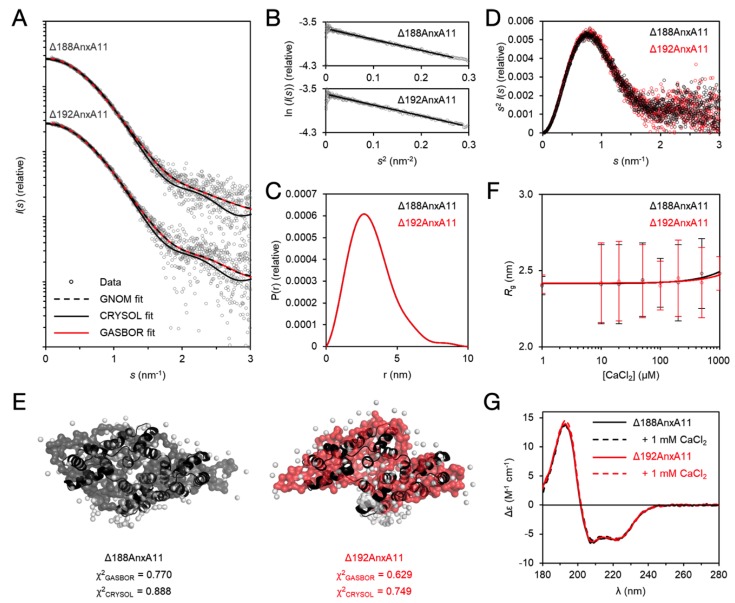
SAXS and SRCD analysis of Δ188AnxA11 and Δ192AnxA11. (**A**) SAXS data for Δ188AnxA11 and Δ192AnxA11 have been offset for clarity. GNOM, GASBOR, and CRYSOL fits are plotted over the measured data to denote the accuracy of distance distribution analysis, ab initio modeling, and theoretical scattering calculated from the crystal structure. (**B**) Guinier analysis. (**C**) Distance distribution diagram from GNOM. (**D**) Kratky plot of the SAXS data. (**E**) GASBOR ab initio models based on the SAXS data. The crystal structure (black) has been fitted inside. χ^2^ values of the GASBOR and CRYSOL fits are indicated. (**F**) R_g_ as a function of CaCl_2_ concentration. The error bars represent the fitting error from Guinier analysis. (**G**) SRCD spectra of Δ188AnxA11 and Δ192AnxA11 in the presence and absence of 1 mM CaCl_2_.

The thermal stability difference between Δ188AnxA11 and Δ192AnxA11 prompted us to study their conformation in solution using SAXS and SRCD. In SAXS, both variants were monomeric with identical distance distributions and globular folds that superposed well with the crystal structure (Figure 7A–E), indicating that the absence of the RGTI stretch does not lead to large-scale conformational changes. Despite the trimeric packing in the crystal, AnxA11 is monomeric in solution under the employed conditions.

Similarly to most Anxs, AnxA11 has been implicated in Ca^2+^ binding [1,13]. Structural studies of many Anxs have revealed Ca^2+^ binding to the convex side of the core structure, essentially through coordination by acidic residues. To test for effect of Ca^2+^ on the folding of the core structure of AnxA11, we titrated Δ188AnxA11 and Δ192AnxA11 with CaCl_2_ in SAXS and followed the radius of gyration (R_g_; Figure 7F). The R_g_ of both truncated proteins remained the same up to 1 mM CaCl_2_. Additionally, we carried out SRCD spectroscopy of Δ188AnxA11 and Δ192AnxA11 in the absence and presence of 1 mM CaCl_2_ (Figure 7G). The spectral quality was excellent, with the data being truncated at 178 nm. The presence of Ca^2+^ did not alter the spectra, indicating that Ca^2+^ does not influence the secondary structure content of the core structure. This result confirms that Δ192AnxA11 is folded despite its dramatic decrease in thermal stability, and that the degree of secondary structure content between the two variants is nearly identical. Ca^2+^ induced a large thermal stabilisation of FL-AnxA11, indicated by a rise in T_m_ of ~14 °C [13]. The presence of up to 75 mM Ca^2+^ only induced small increases in AnxA11 α-helical content [13]. Thus, Ca^2+^ binding results in only local conformational changes, which primes AnxA11 for lipid membrane binding.

## 4. Conclusions

As the diversity of the vertebrate Anx family is mainly realised through the uniqueness of the N-terminal tail [1,16,75,76], many functional features are lost by truncation. However, crucial functional properties of interest, such as mRNA binding, Ca^2+^-dependent binding to phospholipids, as well as Ca^2+^ binding itself, may be retained. Thus, truncated Anxs may serve as a foundation for functional studies, dodging practical issues often encountered due to their long N termini.

The crystal structure of AnxA11, although similar to known Anx core domain structures, provides important starting points to further understand the structure–function relationships in AnxA11 and other Anx family members. The identification of a strongly stabilizing Velcro-like bridging segment at the N terminus of the Anx core, as well as the fact that many known ALS mutations correspond to highly conserved buried Arg residues in the Anx fold, provide new insights into Anx folding. AnxA11, like many other ALS targets, is part of a membraneless organelle, a ribonucleoprotein complex, involved in neuronal transport of mRNA granules [8,77,78,79]. It is evident that this process can be affected by mutations in either the N-terminal region or the folded core domain of AnxA11, which in turn can affect binding to RNA, lipid membranes, and Ca^2+^. The fact that the bridging segment is a target for regulatory phosphorylation in AnxA2 shows that in addition to simply stabilizing the core structure, this segment may be a conformational switch common to most Anxs.

## Figures and Tables

**Table 1:** Small-angle X-ray scattering parameters and analysis.

**Data Collection Parameters**		
Instrument	P12, PETRAIII, DESY	
Wavelength (nm)	0.124	
Angular range (nm^−1^)	0.022–7.33	
Exposure time (s)	0.045	
Exposure temperature (°C)	10	
Protein	AnxA11Δ188	AnxA11Δ192
Concentration range (mg/mL)	0.77–2.96	0.41–1.18
**Structural Parameters**		
*I*_0_ (relative) [from Guinier]	0.02759	0.0274
*R*_g_ (nm) [from Guinier]	2.44 ± 0.13	2.43 ± 0.20
*I*_0_ (relative) [from P(r)]	0.02692	0.02748
*R*_g_ (nm) [from P(r)]	2.500 ± 0.016	2.503 ± 0.019
*D*_max_ (nm) [from GNOM]	10.00	9.97
V_Porod_ (nm^3^) [from GNOM]	58.03	59.53
**Molecular Mass Determination**		
Molecular mass M_r_ (kDa) [from *I*_0_ using Guinier] ^1^	34.3	34.1
Molecular mass M_r_ (kDa) [from *I*_0_ using P(r)] ^1^	33.5	34.2
Molecular mass M_r_ (kDa) [from V_Porod_]	34.1	35.0
Molecular mass M_r_ (kDa) [from absolute scale]	34.8	34.8
Theoretical M_r_ from sequence (kDa)	36.8	36.4
**Software**		
Primary data reduction & processing	PRIMUS	
Ab initio modelling	GASBOR	
χ^2^	0.770	0.629
Fitting of theoretical scattering curve to data	CRYSOL	
χ^2^	0.888	0.749

^1^ Monomeric BSA standard was used as reference (M_r_ = 66.5 kDa; *I*_0_ = 0.0534).

**Table 2:** Crystallographic data collection and refinement.

**Data Collection**	
Construct	∆188AnxA11
Wavelength (Å)	0.9763
Space group	P1
Unit cell parameters	*a* = 39.1 Å, *b* = 86.7 Å, *c* = 87.3 Å ⍺ = 114.0°, β = 102.0°, γ = 97.2°
Resolution range (Å)	50–2.30 (2.36–2.30) ^1^
Completeness (%)	95.4 (95.0)
Reflections total/unique	141110/42245 (9818/3046)
⟨I/σI⟩	5.2 (1.1)
R_sym_ (%)	17.5 (164.0)
R_meas_ (%)	20.9 (196.6)
CC_1/2_ (%)	99.2 (47.6)
redundancy	3.3 (3.2)
**Data Processing**	
R_cryst_/R_free_ (%)	22.8 (26.6)
Rmsd bond length (Å)	0.002
Rmsd bond angle (°)	0.4
MolProbity score (percentile)	1.58 (98th)
Ramachandran favoured/disallowed (%)	97.2/0.3

^1^ Numbers in parentheses refer to the highest-resolution shell.

## Supplementary Materials

The following are available online at https://www.mdpi.com/2218-273X/10/4/660/s1, Figure S1: Δ188AnxA11 and Δ192AnxA11 SAXS dilution data.
