# Supplementary material for: Structure of the ALS Mutation Target Annexin A11 Reveals a Stabilising N-Terminal Segment
Source: Biomolecules. 2020 Apr 24;10(4):660. doi: 10.3390/biom10040660 (PMC7226064; doi:10.3390/biom10040660)
Supplement: Supplementary file 1 [file biomolecules-10-00660-s001.pdf]

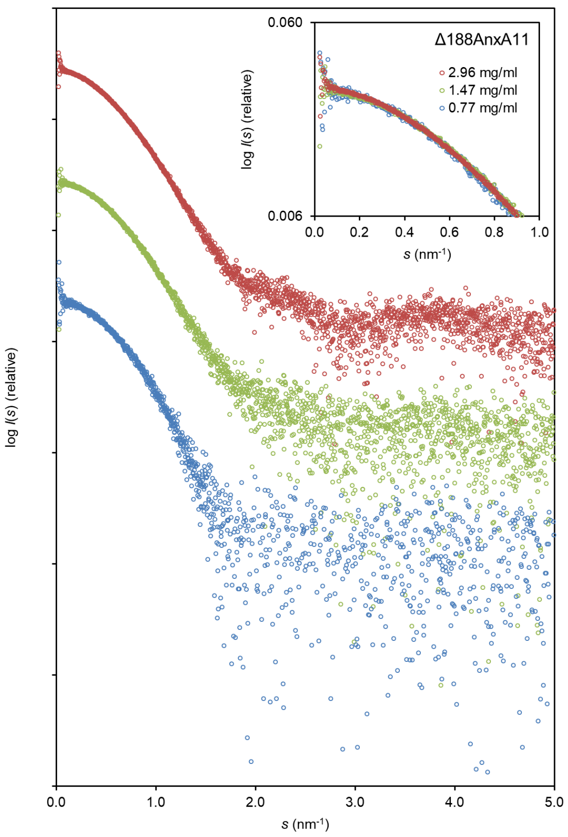

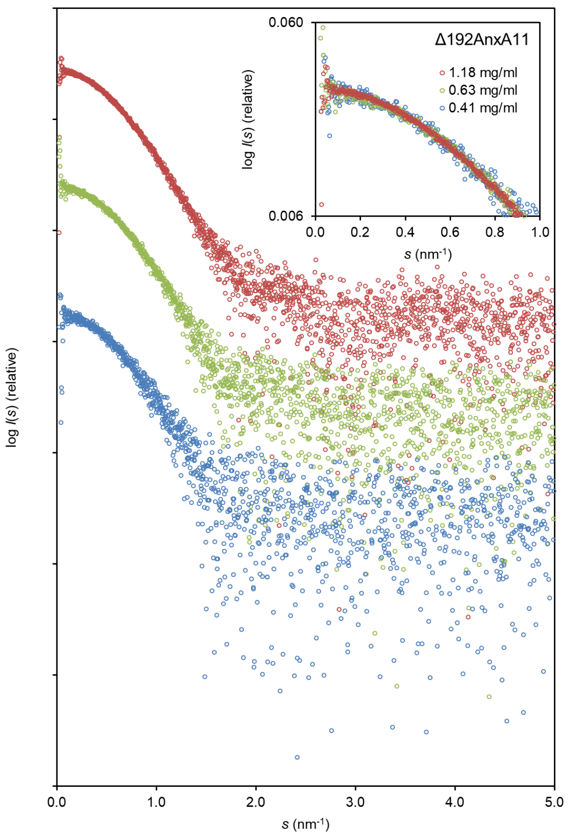

Figure S1. Δ188AnxA11 and Δ192AnxA11 SAXS dilution data. Background-subtracted and concentration-normalised scattering data of Δ188AnxA11 (left) and Δ192AnxA11 (right) are shown. The curves have been offset for clarity. The inset in both graphs shows the different concentrations superposed with one another at low angles, as well as the concentration of each dataset.
